# Etiology of Respiratory Complications among Iranian HIV Infected Patients

**Published:** 2019-02

**Authors:** Majid Marjani, Mahtab Moeinpour, Afshin Moniri, Shadi Khabiri, Seyed Mohammadreza Hashemian, Payam Tabarsi, Ali Akbar Velayati

**Affiliations:** 1 Clinical Tuberculosis and Epidemiology Research Center, National Research Institute of Tuberculosis and Lung Diseases (NRITLD), Masih Daneshvari Hospital, Shahid Beheshti University of Medical Sciences, Tehran, Iran,; 2 Virology Research Center, NRITLD, Masih Daneshvari Hospital, Shahid Beheshti University of Medical Sciences, Tehran, Iran

**Keywords:** HIV, AIDS, Pulmonary, Tuberculosis, Respiratory complications

## Abstract

**Background:**

Infection with Human Immune deficiency Virus (HIV) is a growing problem in developing countries. Among HIV infected cases, respiratory complications are common, dissimilar in different setting and their diagnosis is challenging. The aim of this study was to determine the spectrum of infectious and non-infectious pulmonary complications among HIV infected patients.

**Materials and Methods:**

The retrospective study was done among 710 HIV infected patients admitted in Masih Daneshvari Hospital, National Research Institute of Tuberculosis and Lung Diseases, Tehran, Iran from January 2003 to March 2017. Demographic, clinical, radiologic and laboratory data of 836 episodes of pulmonary complications were reviewed and final diagnosis were extracted.

**Results:**

Mean of CD_4_ cell count was 90±131 ×10^6^ cells/L. Definite etiology was found for 653 episodes (78.1%) of pulmonary complications. Infectious respiratory diseases were clearly more common than non-infectious etiologies, 86.1 and 7.6%, respectively. Pulmonary tuberculosis, as the leading cause, involved 542 cases (64.8%) and *Pneumocystis jiroveci (P. jiroveci)* was the second infectious agent that was found in 111 cases (13.2%). Among non- infectious causes, bronchiectasis and Chronic Obstructive Pulmonary Disease (COPD) exacerbation were on the top of the list, 21 of 64 (32.8%) and 18 0f 64 (28.1%), respectively. Many patients had more than one etiology. *P. jiroveci* had the highest tendency for dual infections (43 episodes).

**Conclusion:**

Pulmonary complications, especially infections are common among HIV cases in Iran, among them tuberculosis is the most common. Respiratory problems may be the first presentation of HIV infection. Clinicians should be aware about the risk of dual infections. Screening for HIV among all tuberculosis cases and vice versa is recommend.

## INTRODUCTION

Human Immune deficiency Virus and Acquired Immune Deficiency Syndrome (HIV/AIDS) continue to be major public health problems in the world. World Health Organization (WHO) estimates 36.7 million people living with HIV worldwide at the end of 2016, 1.8 million of them becoming newly infected and 1.0 million people died from HIV-related causes globally in 2016 (1).

In 2016, number of people living with HIV in Islamic Republic of Iran was estimated 66,000 (37,000 – 120,000), with 5000 (1400 – 13,000) new infections and 4000 (2500 – 6200) AIDS-related deaths. Since 2010, new HIV infections have increased by 21%. The Injecting Drug Users (IDUs) and sex workers comprise the core of HIV epidemic in Iran with an HIV prevalence of 9.3 and 2.1%, respectively (2).

Since the beginning of the HIV epidemic, lung diseases have been a significant cause of morbidity and mortality among HIV-infected individuals (3). About 70% of HIV patients have a pulmonary complication in the course of the disease; most of them are infections (4). For example, lower respiratory tract infections are 25-fold more common in HIV infected cases than in the general population (5).

Development of Antiretroviral Therapy (ART) and decrease in the burden of opportunistic infections has resulted in a shift from infectious to non-infectious pulmonary complications in some setting. Non-infectious complications involve lung airways, parenchyma, and vasculature, such as Chronic Obstructive Lung Diseases (COPD), lung cancer, and pulmonary hypertension (6, 7). It is estimated that up to 25% of HIV infected people may have COPD (8, 9). However, pulmonary infections are still the most frequent cause of admission among HIV cases in resource-limited settings (3, 6).

Spectrum of lung complications occurring in HIV infected individuals, especially infections, is different in diverse geographic regions (10) depending on local epidemiology, efficacy of public health to detect HIV cases in early course of disease and coverage of ART. For example, in Africa, Tuberculosis (TB) is the most common pulmonary complication of HIV (11, 12), but *Pneumocystis jiroveci (P. jiroveci)* Pneumonia (PJP) is uncommon (12, 13). PJP has remained the most frequent opportunistic infection in North America and Europe, whereas pulmonary TB is more common in Eastern Europe (14).

Adequate knowledge about epidemiology and spectrum of the respiratory complications among HIV infected patients helps clinician to apply a better diagnostic and therapeutic approach. So we performed this retrospective study to investigate about infectious and non-infectious etiologies of respiratory problems in HIV cases in Iran.

## MATERIALS AND METHODS

### Study population

This retrospective study was performed in Masih Daneshvari Hospital, National Research Institute of Tuberculosis and Lung diseases (NRITLD), Tehran, Iran. All HIV infected patients admitted in the hospital from January 2003 to March 2017 were recruited. Among them, by using data sheet records and hospital information system, patients with pulmonary complications were selected. Demographic data; records of laboratory studies consisting CD4 lymphocyte cell counts, microbiologic and pathologic investigations, acid-fast staining, molecular investigations for mycobacteria, analysis of pleural fluid, immunohistochemistry studies for pneumocystis, Polymerase Chain Reaction (PCR) for Cytomegalovirus (CMV), results of lung imaging, and echocardiography were reviewed and final diagnosis were extracted. Any admission due respiratory complication was considered as one “episode” and if a patient was re-admitted in the course of recent respiratory disease, latter admissions were not counted. Patients might have more than one etiology during each episode.

Ethical permission for the study was obtained from the Ethics Review Board of NRITLD. The research group was strictly bounded to have particular concern about privacy of the patients under study.

### Definitions

For the purpose of this study, major clinical problems were defined.

Community acquired pneumonia was diagnosed when patients presented with compatible imaging, symptoms and/or positive cultures of bacterial agents. Rule out of TB was necessary.

Empyema was diagnosed by presence of frank pus in the pleural space or detection of micro-organism in the pleural fluid.

Pulmonary TB was confirmed by mycobacteriologic studies. Probable TB was diagnosed by imaging and history compatible with TB without confirmation. Pleural TB was confirmed by 1) positive mycobacteriologic study of sputum and/or pleural fluid; or 2) presence of granuloma in histological study. Diagnosis on the base of Adenosine Deaminase (ADA) level in pleural fluid was considered as probable case. In the patients with pulmonary TB, if another site other than lung and pleura was involved, the episode classified as disseminated TB.

For confirmation of infection with nontuberculous mycobacteria (NTM), the last guideline of American Thoracic Society (ATS) was used (15).

PJP was confirmed by presence of *P. jiroveci* in Bronchoalveolar Lavage (BAL) or lung biopsy specimens identified by special staining or immunohistochemistry assays. In the absence of confirmatory criteria, if clinician decided to initiate specific treatment for PJP on the base of radiologic and clinical setting, it was considered as probable case.

CMV pneumonitis was confirmed by pathologic study. Without pathologic confirmation, if other causes were excluded, lung image was consistent and CMV viremia was detected; it was considered as probable case of CMV pneumonitis.

Pulmonary aspergillosis was confirmed by showing invasive fungal elements in lung tissue. In the absence of confirmation, diagnosis considered probable if 1) fungi were found in lower respiratory specimens and other causes were ruled out or 2) if the BAL fluid galactomannan index was ≥1.

Diagnosis of bronchiectasis was based on compatible radiology and exclusion of other causes especially TB.

Diagnosis of COPD exacerbation was on the base of history, symptoms, imaging and clinical judgment of the physicians that was recorded.

For this study we considered pulmonary hypertension as peak systolic pulmonary arterial pressure equal or more than 35 mm Hg estimated by resting transthoracic echocardiography.

All data were entered into SPSS (version 15.0) for statistical analysis.

## RESULTS

In the period of study 1416 admissions were found in 710 HIV infected patients. Among them, 641 cases had 836 episodes of pulmonary complications resulted in 946 admissions (66.8% of all admissions). Most of HIV cases with pulmonary complication were male (90.6%) and intravenous drug users (69.9%). Mean age of them was 38±9.4 ranged from 1 to 85 years old. CD_4_ lymphocyte cell count ranged from 1 to 884 ×10^6^ cells/L with the mean of 90±131 ×10^6^ cells/L and the median of 40 ×10^6^ cells/L.

About 64% of patients (522 cases) were known cases of HIV, but for 295 patients HIV was found firstly after presentation of pulmonary complications. Only 160 patients were under antiretroviral therapy, 30.6% of all previously known cases of HIV infection. Table 1 shows summary of demographic and basic characteristics of the HIV patients with pulmonary complications.

**Table 1.** Demographic characteristics of 641 HIV infected patients with 836 episodes of respiratory complications

**Parameter**	**N (%)**
**Age** (Mean±SD)	38±9 years
**Sex**	
Male	581 (90.6)
Female	60 (9.4)
Risk factor for HIV infection^*^	
IDU	448 (69.9)
Sexual contact	71 (11.1)
Prison	335 (52.3)
Tattooing	118 (18.4)
Others	2 (0.3)
Unknown	104 (16.2)
**CD_4_ lymphocyte cell count^$^**	
Mean±SD	90±131
<50	368 (57.3)
50–200	198 (30.8)
201–350	42 (6.5)
>350	34 (5.3)
**HIV diagnosis^#^**	
New cases	295 (36.1)
Known cases	522 (63.9)
**ART situation^@^**	
Drug naïve	548 (69.2)
Under ART	160 (20.2)
Drug interruption	84 (10.6)
**History of old TB**	229 (27.4)
**History of PJP**	26 (3.1)

**Abbreviations:** HIV: Human Immune deficiency Virus; SD: Standard Deviation; IDU: Intravenous Drug User; ART: Antiretroviral Therapy; TB: Tuberculosis; PJP: Pneumocystis jiroveci Pneumonia

* Some of patients had two or more risk factors.

$ CD_4_ Count was available in 642 episodes.

# Missing data: 19

@ Missing data: 44

Definite etiology was found for 653 episodes (78.1%) of pulmonary complications, four of them were diagnosed after death. For 131 episodes, diagnosis was probable. Despite meticulous workup, 52 HIV patients (6.2% of all pulmonary cases) didn’t have any diagnosis for their respiratory problems.

Etiologies of pulmonary complications were listed in Table 2. Infectious respiratory diseases were clearly more common than non-infectious etiologies, 86.1 and 7.6%, respectively.

**Table 2.** Etiology of 836 episodes of pulmonary complications among 641 HIV patients^*^

**Etiology**	**Total n(% of all)**	**Definite n(% of all)**	**Probable n(% of all)**
**All Diagnosed**	784 (93.8)	653 (78.1)	131 (15.7)
**Infectious**	720 (86.1)	618	102
**Bacterial**	632 (75.5)	591	41
CAP	41(4.9)	NA	NA
Lung abscess	1 (0.1)	1	0
Aspiration pneumonia	4 (0.5)	NA	NA
Empyema	19 (2.3)	19	0
Septic emboli	7 (0.8)	5	2
VAP	26 (3.1)	26	0
Tuberculosis	542 (64.8)	494	48
Pulmonary	388 (71.5)^$^	355	33
Pleural	15 (2.7)	11	4
Pulmonary and pleural	71 (13)	67	4
Disseminated	68 (12.5)	61	7
Non TB mycobacteria	7 (0.8)	7	0
*M. kansasii*	4 (57.1) ^$^	4	0
*M. Simiae*	1 (14.2)	1	0
*M. abscessus*	1 (14.2)	1	0
*M. gordonae*	1 (14.2)	1	0
Nocardiosis	2 (0.2)	2	0
Actinomycosis	2 (0.2)	2	0
**Fungal**	116 (13.8)	46	70
PJP	111 (13.2)	46	65
Aspergillosis	5 (0.6)	0	5
**Viral**	23 (2.7)	7	16
Influenza	7 (0.8)	2	5
CMV pneumonitis	16 (1.9)	5	11
**Non infectious**	64 (7.6)	63	1
Bronchiectasis	21 (2.5)	NA	NA
COPD exacerbation	18 (2.1)	NA	NA
Pulmonary hypertension	9 (1.1)	9	0
Organizing pneumonia	4 (0.4)	4	0
ARDS	4 (0.5)	NA	NA
Tracheoesophageal fistula	2 (0.2)	2	0
Kaposi sarcoma	2 (0.2)	1	1^#^
Lymphoma	1 (0.1)	1^¥^	0
ILD	1 (0.1)	1	0
Lung fibrosis	1 (0.1)	1	0
Lung cancer	1 (0.1)	1^©^	0
PTE	1 (0.1)	1	0
Cardiopulmonary edema	1 (0.1)	NA	NA
**Others^@^**	19 (2.2)	18	1
**Undiagnosed**	52 (6.2)	-	-

* Some patients had multiple etiologies in one episode. So the sum of the etiologies is higher than the episodes (see text).

$ Percentage of TB or NTM diseases groups

# In both cases, Kaposi sarcoma was confirmed by skin biopsy; for one of them lung biopsy was inconclusive and for another case lung biopsy was not done, but lung infiltration resolved after chemotherapy. After rule out of other causes pulmonary infiltration considered as Kaposi involvement.

¥ Non-Hodgkin lymphoma

© Primary lung adenocarcinoma

@ Others like pneumothorax, rule out of TB without any other diagnosis, etc.

**Abbreviations:** CAP: Community Acquired Pneumonia; VAP: Ventilator Associated Pneumonia; ARDS: Acute Respiratory Distress Syndrome; ILD: Interstitial Lung Disease; PTE: Pulmonary Thromboembolism. NA: Not Applicable (for category counting, NAs were considered as definite diagnosis).

Bacterial agents, especially mycobacteria, were the most common group responsible for pulmonary infections. TB, as the leading cause, involved 542 cases, 64.8% of all episodes of respiratory complications. Pleural involvement and dissemination were found among 15.7 and 12.5% of tuberculosis patients, respectively.

*P. jiroveci* was the second infectious agent of respiratory involvement that found in 111 cases, 13.2% of all episodes of pulmonary complications. Forty six of them (41.4%) were diagnosed definitely and others were treated empirically without confirmation.

Among non-infectious respiratory episodes, bronchiectasis and COPD exacerbation were more common than others, 21 of 64 (32.8%) and 18 of 64 (28.1%), respectively.

Many patients had more than one etiology when they were admitted due to one episode of respiratory complication. Nineteen cases had concurrently infectious and non-infectious problems. Two patients suffered from two non-infectious complications together, and 60 cases (8.3% of cases with infectious complications) had more than one infectious agent in the same time. *P. jiroveci* had the highest tendency for double infections (43 episodes); the most common feature was combination of TB and PJP in 24 patients.

Table 2 also shows details of definite and probable diagnosis for any etiology if it is applicable.

**Figure 1.**
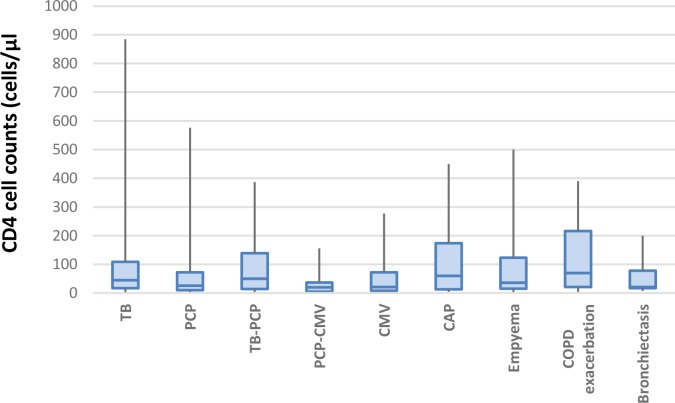
Relationship between CD4 lymphocyte cell count and selected pulmonary complications. The bottom and top of the boxes show the first and third quartiles, and the band inside any box is the median. Ends of the whiskers represent the minimum and maximum of the CD4 for every disease.

Table 3 shows details of immunologic status in various etiologies of pulmonary complications and figure 1 shows distributions of CD_4_ lymphocyte cell counts for selected more common respiratory diseases.

**Table 3.** Immunologic (CD_4_ lymphocyte cell counts) status of various etiologies of pulmonary complications^*^

**Etiology**	**CD_4_**		

	**Range**	**Mean±SD**	**Median**
**CAP**	4–450	124±140	60
**Empyema**	2–500	101±137	36
**VAP**	1–121	27±36	10
**Tuberculosis**	1–884	90±121	45
Pulmonary	2–884	92±124	47
Pleural	6–518	226±198	159
Pulmonary and pleural	1–500	63±84	42
Disseminated	4–387	67±82	40
**Non TB mycobacteria**	5–120	44±48	22
**PJP**	1–576	63±93	63
**Aspergillosis**	13–42	27±15	25
**Influenza**	45–531	206±228	124
**CMV pneumonitis**	1–277	55±79	21
**Bronchiectasis**	7–200	53±61	21
**COPD exacerbation**	4–390	131±134	70
**Pulmonary hypertension**	7–880	179±344	55

* Detail of CD_4_ lymphocyte cell counts was mentioned just for etiologies with number more than 10.

SD: standard deviation

## DISCUSSION

In this retrospective study, patients with respiratory complications with frequency of 66.8% were the largest group among admitted HIV cases. This finding is compatible with other studies (4, 16) that ranked respiratory problems as the most frequent complication of HIV infection (3).

Majority of the respiratory complications had infectious etiology. Among them, TB was the most common (64.8%). Previous studies showed a wide spectrum of incidence for TB among HIV cases. In one study on 599 HIV-infected patients from 1995 to 1998 that only confirmed pulmonary complications were included, *Mycobacterium tuberculosis* (*M. tuberculosis*) was isolated from respiratory specimens of 40 among 1,225 consecutive hospital admissions (3.2%) (17). In a retrospective review of autopsy records, 13 of 233 patients with HIV infection who died between 1985 and 1996 had lung involvement with *M. tuberculosis* (16). Both of these studies were performed in US. Benito found TB in 9 episodes (8.8%) of 102 admitted HIV cases with new onset pulmonary infiltration in Spain (18). In a more recent study by Lee and colleagues in Taiwan, TB was the fourth common infection ranked after PJP, bacterial pulmonary infections and CMV pneumonitis; 17 of 203 respiratory episodes were pulmonary TB (8.4%) (19). In another study from Iran that was performed on 199 new admissions (respiratory and non-respiratory) among HIV-infected patients between 2000 and 2005, pulmonary TB was the most common complication involved 54 of 95 patients with respiratory diseases (56.8%) (20). This rate is close to our results and shows high proportion of pulmonary TB among Iranian HIV cases. On the other hand, in HIV-TB co infected patients, some clinical and diagnostic aspects are different from non HIV patients (21, 22). So, in Iran, clinicians should think about TB in every HIV patient with respiratory problems and exclude it appropriately.

In comparison to other studies (16, 17, 23), frequency of non TB mycobacterial infections was extremely lower than TB in our setting. Furthermore, another study from Iran didn’t report any case with pulmonary NTM disease (20). In our setting, PCR assay for detection of M. tuberculosis was available and clinicians performed it for all patients with TB-HIV co infection as a routine practice of care to rule out NTM. The reason for low incidence of NTM may be high proportion of latent TB infection or higher rate of TB transmission among subgroups of HIV cases, specially IDU and prisoners (24). It is likely that Iranian HIV cases experience active TB before profound collapse of immune system which turns them more susceptible to NTM diseases.

Another aspect of our finding is the prevalence of dual infections. 8.3% of the patients with infectious complications had more than one infectious pathogen in the same time. Respiratory infections with more than one infective agent are common among patients with HIV infection, particularly in the setting of advanced AIDS (3). In one study, 15% of all pulmonary infections were poly microbial (18). Presence of 24 patients with co infection of TB and PJP is remarkable in our study. Castro et al. reported 17 patients who had concurrent pulmonary TB and PJP (25), and one case was reported from Japan (26). Clinicians should be aware that HIV-infected patients may have more than one infectious problem in the lung. They have to consider TB even in the context of confirmed PJP. On the other hand, if clinical setting and radiology are doubtful for PJP, even in the presence of positive results of mycobacterial studies, diagnostic workup for PJP is necessary.

Over the period of our study just 7.6% of respiratory complications were non-infectious. Most of them were cases of bronchiectasis and COPD exacerbations. 69.9% of our patients were active or ex-IDU with probably high frequency of smoking among them, so we expected higher frequency of COPD exacerbation. Many cases of COPD with HIV infection are managed in the periphery and outpatient; this may be the cause of lower frequency of them among admitted patients.

Low incidence of non-infectious problems in comparison to infectious pulmonary complications is not surprising and other authors reported the same results (3, 17–19). Trend of better screening for HIV infection and earlier initiation of ART, reduces the rate of opportunistic infections and improves survival. Consequently, there may be a shift in the epidemiology of pulmonary diseases in HIV infected patients, from infectious to non-infectious and chronic lung diseases (10).

In 36.1% of our patients, respiratory symptoms were the first presentation of HIV infection. This finding shows the importance of HIV case finding among patients with unusual or potentially opportunistic pulmonary infections, especially tuberculosis. WHO recommend screening of HIV among all TB cases and vice versa (27). Mean and median of CD_4_ lymphocyte cell count were very low among our patients, 90 and 40 ×10^6^ cells/L, respectively. It means most of them were in the advanced stage of HIV and AIDS. It shows delay in detection of HIV infection. As the report of UNAIDS, it is estimated that just 38% of people living with HIV in Iran know their status (2). So improvement in screening and case finding is necessary.

Among 836 episodes of respiratory diseases, just 20.2% of them occurred in HIV cases that used antiretroviral drugs. The reminders were drug naïve (69.2%) or interrupted their treatment (10.6%). It may be due to negligence in initiation of antiretroviral therapy and low compliance of patients. In Iran, multiple effective drugs are available free of charge for HIV patients, but just 14% of people living with HIV are on ART (2). Wider coverage of ART for HIV cases and any effort to improve their adherence may decrease the rate of opportunistic infections including respiratory infections.

Good point of our study was availability of suitable facilities for diagnostic workup of pulmonary diseases in our center and meticulous effort for detection of definite etiology. On the other hand, our study has some limitations. The first, it was done in a referral center for TB, lung diseases and HIV. So rate of TB cases may be higher than other settings. On the other hand, patients with milder forms of respiratory complications might be treated in other centers and don't refer to us. It may justify the lower rate of community acquired pneumonia (CAP) among under study patients. Another limitation is the retrospective nature of the study. For better understanding about spectrum of pulmonary diseases among HIV cases in Iran, a large, multicenter, and preferably prospective study is necessary.

## CONCLUSION

Pulmonary complications are common among HIV cases in Iran. It includes infectious and in the lesser part non-infectious problems. Among them TB is the most common. Respiratory diseases, especially opportunistic infections may be the first presentation of HIV infection. Clinicians should be aware about the risk of dual infections. To decrease pulmonary complications among HIV infected patients, improvement of HIV infection case finding and better coverage of ART for known cases is recommended.
